# Striving to Be Thin: Weight Pressures and Eating Disorder Symptoms in Professional Young Female Modern Dancers

**DOI:** 10.3390/nu18121910

**Published:** 2026-06-12

**Authors:** Anastasia Donti, Ioli Panidi, Gregory C. Bogdanis, Dimitra-Anastasia Kanna, Vasiliki Gaspari, Olyvia Donti

**Affiliations:** School of Physical Education & Sport Science, National & Kapodistrian University of Athens, 17237 Athens, Greece; adonti@phed.uoa.gr (A.D.); ipanidi@phed.uoa.gr (I.P.); gbogdanis@phed.uoa.gr (G.C.B.); dimitrakanna@phed.uoa.gr (D.-A.K.); vgaspari@phed.uoa.gr (V.G.)

**Keywords:** dance, eating behaviours, diet, bulimia, anorexia, appearance, performance

## Abstract

**Background/Objectives**: This study examined weight pressures and symptoms of eating disorders in professional modern dancers. **Methods**: Eighty-six female dancers (age: 20.7 ± 2.5 y, dancing experience: 14.2 ± 4.4 y) completed the Eating Attitudes Test 26 (EAT-26), the Weight-Pressures in Sport inventory for females (WPS-F), and provided information on their dance lessons. **Results**: Twenty-five dancers (29.07%) scored ≥20 in EAT-26. Positive associations were found between EAT-26 and its subscales with WPS-F and its subscales (*r* = 0.217 to 0.600, *p* ˂ 0.05). Negative associations were found between age and dancing experience with the EAT-26 score and its subscales Dieting and Bulimia and Food Preoccupation (*r* = −0.286 to −0.373, *p* ˂ 0.001) and between body weight and BMI with Oral Control (*r* = −0.300 to −0.372, *p* ˂ 0.001). Multiple regression analysis showed that Pressures regarding Appearance and Performance, age, and dancing experience accounted for 38.3% of the variance in EAT-26 (*p* ˂ 0.001), with age and training experience showing a negative coefficient. Moderation analysis showed that dancing experience moderates the relationship between Pressures regarding Appearance and Performance and eating disorder symptoms (interaction b = −0.329, *p* = 0.040). **Conclusions**: Professional female dancers are at elevated risk for disordered eating. Inherent pressures regarding appearance and performance were associated with and explained a significant portion of the variance in eating disorder symptom scores, while dancing experience appeared to attenuate this association, although the cross-sectional design of this study precludes conclusions regarding the direction of this effect.

## 1. Introduction

Dance occupies a unique and multifaceted space between art forms, being regarded as both an artistic expression and a sport [1]. Technical precision, physical fitness, and aesthetic expression are key performance determinants that enable the communication of emotion, ideas, and narrative through movement [2]. A dancer’s body is the main “instrument” for reaching the audience; thus, a lean physique is often perceived as necessary to fulfil aesthetic demands and optimise the ratio of power generation relative to body mass [3]. For example, a lean body gives an advantage in performing complex jumping skills and makes it easier to balance in “pointe” while executing multiple turns [4].

The “ideal” body for dance is subject to strict, sometimes excessive demands on controlling body weight and shape [3,5,6]. Dancers often face pressure to maintain a lean physique from an early age, a demand reinforced throughout their careers by teachers, choreographers, and audience expectations, frequently contributing to heightened body dissatisfaction [5,7]. These pressures are further intensified by continuous evaluation, perpetual exposure to mirrors during lessons, and frequent weigh-ins that enhance body hyperawareness and increase the likelihood of unhealthy weight-control practices and ultimately of disturbed eating behaviours [7]. Body image, defined as an individual’s perception, thoughts, and feelings about their own body, plays a central role in this process [8]. In dance contexts, the internalisation of an ideal, slender body often leads to negative body image and body dissatisfaction, both consistently identified as risk factors of disordered eating [9]. This dissatisfaction, often coupled with an intense fear of weight gain, creates a cognitive and emotional environment that leads to the development of eating pathology [9] and may also trigger dietary restriction, compulsive exercise, and other weight-control behaviours driven by the need to conform to perceived aesthetic standards [10].

Eating disorders are severe mental disorders with mortality rates among the highest of all psychiatric illnesses [11,12]. They typically emerge during puberty [13] and last an average of six years, with many individuals remaining undiagnosed and untreated for much of that time [14]. Evidence suggests that genetics and heredity may contribute to heightened susceptibility to eating disorders, but these disorders can also affect those with no family history [15].

Eating disorders exist along a continuum, ranging from optimal nutritional practices to clinically diagnosed eating disorders, with disordered eating representing an intermediate state [16]. Anorexia nervosa and bulimia nervosa represent the most severe forms of disordered eating [17]. Subclinical manifestations, such as excessive preoccupation with body weight and shape, restrictive eating, chronic dieting, binge eating, self-induced vomiting, and the misuse of diuretics or laxatives, can adversely affect health and increase the risk of developing clinical eating disorders later in life [17,18].

Elevated rates of disordered eating are consistently reported in professional compared to lower-level dancers and the general population and in classical dancers compared with dancers of other dance forms, driven by appearance and performance demands [19,20]. In some cases, dancers’ pursuit of leanness to enhance performance leads to food restriction with adverse effects on their menstrual cycle, bone and cardiovascular health, immunity, and overall well-being [19]. However, despite the intense pressures for a “thin ideal” in professional dance, the potential link of weight pressures to eating disorder symptoms, especially among professional modern dancers, has not been explicitly studied. In addition, the source of weight-related pressures in the development of eating disorder symptoms and the potential mediating role of factors such as dancing experience have not been sufficiently addressed. Thus, the aim of this study was to examine the association between eating disorder symptoms and weight pressures in professional modern dancers, with a focus on identifying the sources of weight-related pressures originating from teachers, choreographers, peers, or inherent dance standards. Furthermore, the study sought to contribute to the existing literature on modern dancers, a population subjected to equally demanding, aesthetic, and performance standards as their classical counterparts.

## 2. Materials and Methods

### 2.1. Participants

Eighty-six professional female modern dancers (age: 20.7 ± 2.5 years) participated in this study. The dancers were graduates of professional dance schools, randomly recruited from three different dance schools. Specifically, from the total roster of eligible professional dancers enrolled in each school, participants were selected using simple random sampling, ensuring that every eligible dancer had an equal chance of being included in the study. Dancers practiced four to six times per week, for approximately 2.5 to 4.5 h per day, and participated in dance performances 3–7 times a year.

The study questionnaire consisted of three parts: (a) a section including age, dancing and performance details such as dancing experience, number of dancing lessons per week and number of performances per year (b) the Eating Attitudes questionnaire [EAT-26] [21] and, (c) the Weight Pressures in Sports-Females questionnaire [WPS-F] [22,23]. The questionnaires were distributed to the participants in their dancing facilities, one hour before their dance practice or lesson. Dancers were gathered in small groups in a separate room, with no teachers present. A member of the research team was present during questionnaire completion to provide clarification if needed and to ensure that participants remained focused and completed the inventories independently. Furthermore, dancers were informed about the confidentiality and anonymity of their responses and their right to withdraw from the study at any time without being required to provide any explanation for their decision. Participants responded to the questionnaire on paper and a researcher collected the complete questionnaires.

Body Mass Index (BMI) was calculated as the ratio of body mass to squared standing height (kg/m^2^), based on self-reported values. The characteristics of the participants are shown in Table 1. Prior to the study, the dancers were fully informed about the purpose and procedures of this study and gave written informed consent. Instructions to the participants included a reminder to respond to all items and a statement that there were no right or wrong answers. Procedures were approved by the local Institutional Ethics Review Committee (1645/15-05-2024) and followed the Code of Ethics set by the World Medical Association.

### 2.2. Study Measures

#### 2.2.1. Eating Attitudes Test-26

Participants’ disturbances in eating attitudes and behaviours were evaluated via the Eating Attitudes Test-26 [EAT-26] [21], as adapted for the Greek population [21]. EAT-26 is a self-report questionnaire consisting of 26 questions. The score of each question ranges from 0 to 3 (3 = often, 2 = usually, 1 = always, 0 = sometimes, rarely, or never), and the final score of the questionnaire ranges from 0 to 78. The questionnaire consists of three subscales: (a) Dieting (13 items) (being preoccupied with thinness and avoiding fattening foods), (b) Bulimia and Food Preoccupation (6 items) (thinking about food and bulimic symptomatology), and (c) Oral Control (7 items) (self-control of eating and perceived pressures from others to gain weight) [21]. A previous study on the validity and reliability of the Greek version of the questionnaire demonstrated adequate psychometric properties of the inventory for Greece’s athletic population [21]. Although the EAT-26 questionnaire is not a diagnostic tool for eating disorders, it has been found to be particularly effective in detecting possible cases of eating disorder symptoms, suggesting further investigation for those with a score equal to or higher than 20. In this study, Cronbach’s value for EAT-26 was 0.76.

#### 2.2.2. Weight Pressures in Sports—Females

The Weight Pressures in Sports—Females (WPS-F) Questionnaire [22,23] is an 11-item self-report questionnaire designed to assess the pressures that come from the sport environment and are experienced by female athletes regarding their weight, body shape, size, and appearance. The questionnaire assesses the pressures in women’s sport to maintain a low body weight, coming from four different directions: (a) coaches/teammates/the ‘nature’ of the sport, (b) the athlete’s image and weight consciousness, (c) the importance of outward appearance, and (d) weight restriction (e.g., weight limits). It consists of 11 questions scored on a 6-point Likert scale from 1 (never) to 6 (always). These questions comprise two factors: the Pressures from Coaches and Sports about Weight (6 questions) and the Pressures regarding Appearance and Performance (5 questions). The total score of the questionnaire is obtained by averaging the scores of all 11 questions. Higher values in the questionnaire score mean higher weight pressure in female athletes [22,24]. The inventory has been translated into Greek and validated in Greece’s female athletic population [25]. In this study, Cronbach’s value for the questionnaire was 0.87.

### 2.3. Statistical Analysis

Data were examined for normality, using the Shapiro–Wilk test, with no violations. Data are presented as means and standard deviations for all variables. Pearson’s correlation coefficient (*r*) was used to assess linear associations among EAT-26 scores and the examined variables. To enable comparability across subscales, given that the three EAT-26 subscales contain different numbers of items, item-level means were calculated by dividing the raw subscale score by the number of items in each subscale, and percentage-of-maximum scores were calculated by dividing the raw subscale score by the maximum possible score and multiplying it by 100. Stepwise multiple regression analysis (backward elimination) was used to investigate which of the two subscales of the Weight Pressures in Sports—Females inventory contributed most significantly to the EAT-26 score. Dancers’ age and dancing experience were also included in the multiple regression analysis, as they are important factors for disturbed eating attitudes. Moreover, a moderation analysis was conducted to test whether dancing experience moderates the relationship between Pressures regarding Appearance and Performance and eating disorder symptoms. The analysis was performed using hierarchical regression with an interaction term between dancing experience and pressures entered in the final step to test the moderating effect. All continuous predictors were mean-centred prior to computing the interaction term, and the change in explained variance (ΔR^2^) associated with the interaction was examined. Simple-slope analyses were conducted to probe the interaction at low (−1 SD), mean, and high (+1 SD) levels of dancing experience. Collinearity diagnostics were examined through the Variance Inflation Factor (VIF) and Tolerance indices to assess the degree of multicollinearity among the predictor variables.

An a priori power analysis was conducted using G*Power 3.1 [26] to determine the required sample size. For the multiple regression analysis, based on a large effect size (f^2^ = 0.62), an alpha level of 0.05, a power of 0.80, and three predictors, the minimum required sample size was calculated as *N* = 22. For the correlation analyses, based on a medium effect size (*r* = 0.30), an alpha level of 0.05, and a power of 0.80, the minimum required sample size was calculated as *N* = 64. The present sample of 86 participants exceeded both requirements, ensuring adequate statistical power for all analyses.

Statistical significance was accepted at *p* < 0.05. All hypotheses tested were two-tailed. Analyses were performed using SPSS (version 20.0, SPSS Inc., Chicago, IL, USA).

## 3. Results

A total of 25 female dancers (29.07%) scored ≥20 on the EAT 26. Participants’ answers to the inventories are presented in Table 2.

The item-level means for the three EAT-26 subscales were as follows: Dieting (M = 0.66, SD = 0.59), Bulimia and Food Preoccupation (M = 0.36, SD = 0.49), and Oral Control (M = 0.43, SD = 0.47), indicating that the Dieting subscale yielded the highest item-level mean across the three subscales. The percentage of maximum scores for the three EAT-26 subscales were 22.12% for Dieting, 12.08% for Bulimia and Food Preoccupation, and 14.29% for Oral Control, indicating that all three subscales scored relatively low in relation to their maximum possible scores, with the Dieting subscale yielding the highest percentage among the three.

Significant associations were found between EAT-26 and its subscales with WPS-F and its subscales (*p* ˂ 0.05) (Table 2). A negative association was found between age and dancing experience and EAT-26 total score and its subscales Dieting and Bulimia and Food Preoccupation (*p* ˂ 0.001). Similarly, body mass and BMI were negatively associated with Oral Control (*p* ˂ 0.001) (Table 3).

Multiple regression analysis showed that Pressures regarding Appearance and Performance, age, and dancing experience accounted for 38.3% of the variance in EAT-26 (adjusted R^2^ = 0.382, F = 6.786, *p* ˂ 0.001) (Table 4).

Collinearity statistics indicated no multicollinearity concerns, with tolerance values ranging from 0.882 to 1.000 and VIF values ranging from 1.048 to 1.134. The correlation between age and dancing experience was low (*r* = 0.247, *p* = 0.05; Table 3), further confirming the absence of multicollinearity and supporting the independent contribution of each variable in the model.

Moderation analysis showed that dancing experience significantly moderates the relationship between Pressures regarding Appearance and Performance and eating disorder symptoms (ΔR^2^ = 0.44, interaction β = −0.329, and *p* = 0.040) (Table 5).

**Figure 1:**
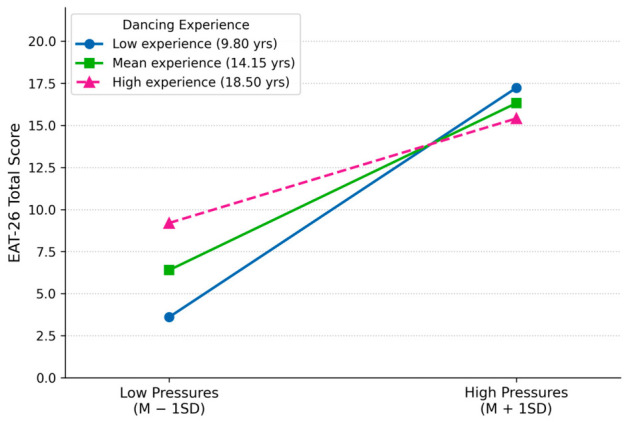
Simple slopes that illustrate the moderating effect of dancing experience on the relationship between appearance/performance weight pressures and eating disorder symptom scores (EAT-total). Interaction b = −0.33, 95% CI [−0.663, −0.040], *p* = 0.040.

In addition, simple slopes illustrating the relationship between appearance and performance pressures and EAT-26 scores at three levels of dancing experience (low: M − 1 SD; mean: M; high: M + 1 SD) are presented in Figure 1.

Figure 1 was constructed with AI-assisted tools based on author-specified parameters (means, SDs and interaction terms).

## 4. Discussion

This study examined the association between eating disorder symptoms and weight pressures in professional modern dancers. Twenty-nine percent of professional female dancers were identified as being at elevated risk for disordered eating. Inherent pressures concerning appearance and performance were associated with and explained a significant portion of the variance in eating disorder symptom scores, while dancing experience appeared to attenuate this association, although the cross-sectional design of this study precludes conclusions regarding the direction of this effect.

Given the cross-sectional design, reliance on self-report measures, and the use of an exploratory data-driven regression approach, the findings of the present study should be interpreted as preliminary and exploratory rather than confirmatory. Accordingly, the observed associations should be viewed as generating hypotheses and informing future research rather than establishing causal or protective mechanisms. Previous studies have primarily examined eating disorder symptoms in ballet dancers, with professional modern dancers being relatively under-researched [17,27]. In the present study, 29.07% of modern dancers scored above the threshold for eating disorder symptoms on the EAT-26, a rate substantially higher than that reported in the general population [27] and exceeding those reported in other studies of modern dancers [28]. Previous research reported that 12.2% of modern dancers scored ≥20 on the EAT-26, and 24.4% of ballet dancers, suggesting a lower risk of eating disorder symptoms in modern dancers [29]. This lower risk is partly attributed to cultural differences among dance styles—for example, ballet’s excessive emphasis on a thin physique and form-fitting attire contrasts with the looser clothing and comparatively reduced focus on leanness in styles such as modern, musical theatre, and hip-hop [30]. Nevertheless, these factors represent only a subset of the many influencing body ideals in dance and may not be the most central. A meta-analysis of cross-sectional studies [19] reported a prevalence of 12% in all dancers and 16.4% among ballet dancers. Notably, in that study, another 14.9% of all dancers demonstrated eating disorders not otherwise specified, and 4% anorexia, resulting in 25% of the dancers being at elevated risk for disordered eating [19]. In an earlier study, the prevalence estimates for any eating disorder ranged from 0 to 45.5% in university-level female modern dancers, while no differences were detected between dance styles in any of the three original subscales of the EAT 26 [31]. The risk among dancers appears to differ based on dance form and performance level, with the latter likely being a primary contributing factor. Eating disorder symptoms are more prevalent among elite female athletes than their recreational peers [17], suggesting that performance level may play a larger role than dance style in their development. Even in modern dance schools, where diverse body types are more accepted, dancers with non-traditional physiques often face limited opportunities to advance to soloist, principal, or professional company roles, underpinning how performance demands can shape both body image and eating behaviours.

The relatively low percentage of maximum scores across all three EAT-26 subscales (Dieting: 22.12%, Bulimia and Food Preoccupation: 12.08%, Oral Control: 14.29%) suggests that while eating-attitude concerns are present in this sample, they do not reach levels indicative of severe pathology. Notably, the Dieting subscale yielded the highest percentage of maximum score, followed by Oral Control and Bulimia and Food Preoccupation. This pattern is consistent with previous findings in dance populations, where dietary restraint and weight control behaviours tend to be more prominent than bulimic behaviours [19]. It should be noted, however, that in a professional dance context, elevated Dieting scores may not exclusively reflect pathological behaviour. Rather, some items may capture nutritional awareness, performance-related dietary monitoring, or occupational knowledge, introducing ambiguity in the interpretation of this subscale. Therefore, elevated Dieting scores in the present sample should be interpreted with caution and understood as possible indicators of eating-attitude concerns rather than clinical pathology.

Significant positive associations were found between EAT-26 total score and its subscales Dieting and Bulimia and Food Preoccupation, with the total score of WPS-F and its subscales. Pressure to lose weight is a common contributor to eating disorders in dancers [32,33]. To align with perceived performance and aesthetic ideals, dancers may feel compelled to reduce their weight through extreme dieting, excessive exercise, or other maladaptive behaviours [34]. Notably, Oral Control was not associated with weight pressures but showed a negative association with body mass and BMI. Oral Control measures self-control related to eating behaviours and perceived social pressure to eat or gain weight. The higher scores among thinner dancers found in this study suggest that thin dancers try harder to control their food intake while feeling more pressure to eat or gain weight. Along this line, the average BMI of female modern dancers in this study was 19.8 kg/m^2^, but in 20 of them (23.26%), the BMI was below 18.5 kg/m^2^ (limit for underweight).

The results of this study indicated that Pressures Regarding Appearance and Performance, age, and dancing experience explained a significant portion of the variance of the EAT 26 score in professional modern dancers. This suggests that both inherent dance pressures and dancers’ maturity and experiences in the dance are associated with eating disorder symptoms in professional modern dancers, albeit age and dancing experience were negatively correlated with symptom scores. This may indicate that older and more experienced dancers may be at lower risk. However, given the cross-sectional design of the present study, no causal or directional inferences can be drawn from these associations. There are numerous unique pressures within the sport and dance environment that may contribute to the development of disordered eating attitudes and behaviours, including the revealing nature of the uniform, the perception of performance advantages gained at a certain weight or body size/shape, weight requirements, and frequent weigh-ins [25]. For example, almost all (99%) in a sample of college female dancers experienced negative body image and feelings of self-consciousness from costumes that failed to hide perceived bodily flaws, and admitted that costumes that were perceived to make them appear unattractive were a performance distraction [6]. Reel et al. [6] in the development of WPS-F also found that the pressures athletes experience in the sport environment about appearance and performance were more strongly related to the measures of general socio-cultural pressures about body, weight, appearance and internalisation, body satisfaction, dietary intent, and bulimic symptoms than the factor regarding coaches as a source of weight-related pressures. Reel et al. [6] assumed that direct comments have a negative effect, but these comments have the strongest influence when an athlete feels negative about her body. One possibility is that athletes who participate in aesthetic sports and dancers become desensitised to comments about weight when they are constantly exposing their bodies (and perceived imperfections) [23]. Along this line, this study also found that dance pressures (Pressures regarding Appearance and Performance) contribute uniquely to explaining dancers’ disturbed eating behaviours. These findings support Petrie and Greenleaf’s (2009) [18] model regarding the aetiology of disordered eating among athletes that the internalisation of sport-related weight pressures would contribute to body dissatisfaction and dietary intent among female athletes. Thus, inherent dance pressures represent a salient feature for female dancers who may be expected to change their weight, shape and size.

An important finding of this study was that age and dancing experience were negatively associated with and predicted the EAT 26 score. Furthermore, moderation analysis showed that dancing experience moderated the relationship between Pressures regarding Appearance and Performance and eating disorder symptoms. Evidence suggests that age may be a protective factor against eating pathology in sports [35]. For example, adolescent and younger adults are typically reported to be at higher risk for eating disorder symptoms than older populations [36], and anorexia nervosa was primarily thought to be seen in people aged between 16 and 25 years [37], and diagnosis could only be made in people aged under 30 years [38]. Thus, it seems that age can be a protective factor against eating pathology, because older individuals often develop greater body acceptance and resilience to societal or performance-related appearance pressures. Notably, in this study, dance experience appears to buffer the association between inherent pressures within the dance environment and eating disorder symptoms. This protective effect may be attributed to experienced dancers’ enhanced ability to cope with both performance-related pressures and associated emotional demands, which in turn may contribute to more adaptive regulation of eating habits. Nevertheless, the apparent protective association of dancing experience may reflect selection effects rather than a genuine protective mechanism. For instance, the younger dancers in this sample had already accumulated considerable dancing experience (~14.5 y), and it is plausible that dancers with greater eating-attitude concerns may have withdrawn from professional pathways earlier. This would result in a more experienced surviving cohort that appears more resilient, potentially inflating the perceived protective effect of experience.

Despite the contribution of the present study in examining the association between eating disorder symptoms and weight pressures in professional modern dancers, there are some limitations that should be reported. Data in this study were collected from self-report instruments. While inventories offer practical benefits, including ease of use and preliminary insights, this study did not include clinical interviews, dietary-intake data, physiological indicators, energy-availability measures, menstrual or bone-health data, or objective training-load measures. Therefore, the findings cannot establish whether participants were under-fueling, experiencing clinically diagnosed eating disorders, or showing physiological consequences of disordered eating. The elevated scores observed should be interpreted as indicators of eating-attitude concerns and possible disordered eating risk rather than clinical diagnoses, and an accurate diagnosis would require validation through clinical interviews and comprehensive diagnostic assessments. Similarly, body mass index was derived from self-reported body mass and height, which may be subject to reporting bias, as individuals tend to underestimate weight and overestimate height, potentially affecting the accuracy of BMI classifications. Another limitation is the small sample size of professional modern dancers, which restricts the statistical power to detect small or medium effect sizes and may introduce bias. Furthermore, the use of backward stepwise regression, although appropriate for the exploratory nature of the present study, is a data-driven approach that may produce unstable results; future studies should employ hierarchical regression with theoretically justified entry of predictors. Nevertheless, this study reveals a novel insight: performance demands and inherent dance pressures are associated with eating disorder symptoms, yet dancers appear to develop—at least to some degree—more effective strategies over time. The findings suggest that with accumulated professional experience, the association between weight-related and performance pressures and dancers’ eating behaviours may be attenuated.

Diagnosing eating disorder symptoms in dancers can be challenging due to denial, a sense of control, and secrecy [39,40,41]. The worrying signs commonly seen in the general population may be masked in the dancing environment. For example, strict dietary habits and preconceived notions of weight and body shape may reflect the customary practices of sports, low body fat may be balanced by increased muscle mass resulting in a normal body weight, and excessive physical activity may be justified by the demands of training [41].

## 5. Conclusions

Inherent perceived pressures related to appearance and performance were associated with and explained elevated self-reported eating-attitude concerns in this sample of professional modern dancers. Age and dancing experience may reduce the severity of eating disorder symptoms; however, the cross-sectional design of this study precludes conclusions regarding the direction of this effect. The present study provides preliminary evidence and a rationale for more rigorous future investigation. Future research should employ longitudinal designs with theoretically specified models and clinical or behavioural outcome measures. Potential mechanisms warranting examination include coping strategies, body image, resilience, energy availability, and training load, all of which may contribute to eating disorder risk in professional dancers.

## Figures and Tables

**Table 1:** Anthropometric characteristics and dance performance parameters of the participants (means ± SD).

	Professional Modern Dancers (*n* = 86)
Age (y)	20.7 ± 2.5
Dancing experience (y)	14.15 ± 4.5
Height (cm)	162.0 ± 5.5
Body mass (kg)	52.4 ± 4.1
BMI (kg/m^2^)	19.8 ± 1.4
*Dance performance*	
International performances per year	1–2
National performances per year	3–5
Hours of dancing lessons per week	18–30
Number of dancing classes per week	4–6

**Table 2:** Answers of the participants in the items of the inventories (means ± SD).

	Professional Modern Dancers (*n* = 86)
EAT-26	13.80 ± 10.41
*Diet*	8.63 ± 7.65
*Bulimia and Food Preoccupation*	2.17 ± 2.91
*Oral Control*	3.00 ± 3.32
WPS-F	6.65 ± 2.10
*Pressures from Coaches and Sports about Weight*	3.23 ± 1.20
*Pressures regarding Appearance and Performance*	3.42 ± 1.21

**Table 3:** Associations between the examined variables.

	1	2	3	4	5	6	7	8	9	10	11
1	1										
2	0.247 *	1									
3	0.083	−0.075	1								
4	0.156	0.064	0.511 **	1							
5	0.102	0.128	−0.373 **	0.605 **	1						
6	−0.373 **	−0.286 **	−0.076	−0.012	0.052	1					
7	−0.301 **	−0.362 **	−0.109	−0.026	0.076	0.419 **	1				
8	−0.016	0.062	0.044	−0.300 **	−0.372 **	0.230 *	−0.009	1			
9	−0.363 **	−0.292 **	−0.072	−0.112	−0.060	0.926 **	0.585 **	0.486 **	1		
10	−0.210	−0.014	−0.125	0.131	0.249 *	0.600 **	0.217 *	0.107	0.536 **	1	
11	0.066	−0.113	−0.084	0.036	0.117	0.311 **	0.146	0.093	0.299 **	0.510 **	1
12	−0.083	−0.073	−0.121	0.096	0.211	0.525 **	0.209	0.115	0.481 **	0.870 **	0.868 **

*: *p* ≤ 0.05, **: *p* ≤ 0.01 **Note**: 1: age, 2: dancing experience, 3: height, 4: body mass, 5: BMI, 6: Dieting, 7: Bulimia and Food Preoccupation, 8: Oral Control, 9: Eat-26 total score, 10: Pressures regarding Appearance and Performance, 11: Pressures from Coaches and Sports about Weight, 12: WPS-F total score.

**Table 4:** Results of the multiple regression analysis predicting Pressures regarding Appearance and Performance from dancers’ age and dancing experience (*n* = 86).

	B	*SEB*	β	*p*	95% CI for B	
					Lower Bound	Upper Bound
Step 1						
Constant	−1.972	2.871			−7.682	3.738
Pressures regarding Appearance and Performance	4.614	0.792	0.536	<0.001	3.039	6.190
Step 2						
Constant	7.752	4.038			−0.280	15.785
Pressures regarding Appearance and Performance	4.580	0.751	532	<0.001	3.087	6.073
Dancing experience	−0.679	0.208	−0.284	0.002	−1.093	−0.264
Step 3						
Constant	25.013	8.630			7.845	42.180
Pressures regarding Appearance and Performance	4.220	0.750	0.491	<0.001	2.728	5.713
Dancing experience	−0.561	0.210	−0.235	0.009	−0.979	−0.143
Age	−0.857	0.381	−0.202	0.027	−1.615	−0.099

**Table 5:** Moderation analysis testing the moderation effect of dancing experience on the association between Pressures regarding Appearance and Performance and eating disorder symptoms.

	B	*SEB*	β	*p*	95% CI	
					Lower Bound	Upper Bound
Step 1						
Constant	14.086	0.954			12.189	15.982
Pressures regarding Appearance and Performance (centred)	4.614	0.792	0.536	<0.001	3.039	6.190
Step 2						
Constant	14.11	0.904			12.314	15.909
Pressures regarding Appearance and Performance (centred)	4.580	0.751	532	<0.001	3.087	6.073
Dancing experience (centred)	−0.679	0.208	−0.284	0.002	−1.093	−0.264
Step 3						
Constant	13.881	0.888			12.114	15.648
Pressures regarding Appearance and Performance (centred)	4.220	0.750	0.491	<0.001	2.728	5.713
Dancing experience (centred)	−0.561	0.210	−0.235	<0.009	−0.979	−0.143
Age (centred)	−0.857	0.381	−0.202	0.027	−1.615	−0.099
Step 4						
Constant	13.572	0.905			11.770	15.374
Pressures regarding Appearance and Performance (centred)	3.894	0.754	0.453	<0.001	2.393	5.395
Dancing experience (centred)	−0.602	0.218	−0.252	0.007	−1.036	−0.168
Age (centred)	−0.998	0.433	−0.236	0.024	−1.859	−0.137
Interaction_dancing experience	−0.329	0.158	−0.181	0.040	−0.642	−0.015
Interaction_age	−0.387	0.337	−0.111	0.255	−1.057	0.284

## Data Availability

Data are published in the Figshare depository: https://figshare.com/articles/dataset/Donti_2025_data_dancers_weight_pressures_xls/30112282?file=57915346 (accessed on 12 September 2025). Dataset posted on 12 September 2025.

## Abbreviations

The following abbreviations are used in this manuscript:

EAT-26	Eating Attitudes Test-26
WPS-F	The Weight Pressures in Sports—Females Questionnaire
